# Supplementary material for: SARS-CoV-2 induces a durable and antigen specific humoral immunity after asymptomatic to mild COVID-19 infection
Source: PLoS One. 2022 Jan 12;17(1):e0262169. doi: 10.1371/journal.pone.0262169 (PMC8754314; doi:10.1371/journal.pone.0262169)
Supplement: Attachment — Submitted filename: Response to Reviewers.docx [file pone.0262169.s001.docx]

Please include the following items when submitting your revised manuscript:

- A rebuttal letter that responds to each point raised by the academic editor and reviewer(s). You should upload this letter as a separate file labeled 'Response to Reviewers'.
- A marked-up copy of your manuscript that highlights changes made to the original version. You should upload this as a separate file labeled 'Revised Manuscript with Track Changes'.
- An unmarked version of your revised paper without tracked changes. You should upload this as a separate file labeled 'Manuscript'.

Journal Requirements:

When submitting your revision, we need you to address these additional requirements.

1. Please ensure that your manuscript meets PLOS ONE's style requirements, including those for file naming. The PLOS ONE style templates can be found at

<https://journals.plos.org/plosone/s/file?id=wjVg/PLOSOne_formatting_sample_main_body.pdf> and

<https://journals.plos.org/plosone/s/file?id=ba62/PLOSOne_formatting_sample_title_authors_affiliations.pdf>

We have ensured that the manuscript adheres to these style requirements.

2. We note that you have indicated that data from this study are available upon request. PLOS only allows data to be available upon request if there are legal or ethical restrictions on sharing data publicly. For more information on unacceptable data access restrictions, please see <http://journals.plos.org/plosone/s/data-availability#loc-unacceptable-data-access-restrictions>.

In your revised cover letter, please address the following prompts:

a) If there are ethical or legal restrictions on sharing a de-identified data set, please explain them in detail (e.g., data contain potentially sensitive information, data are owned by a third-party organization, etc.) and who has imposed them (e.g., an ethics committee). Please also provide contact information for a data access committee, ethics committee, or other institutional body to which data requests may be sent.

As these data contain sensitive personal information, and the informed consent given by the study participants does not cover sharing the information to third parties (Swedish Ethical Review Authority approval nr 2020-01653), we cannot share this data publicly. However, the data is available on request at https://doi.org/10.17044/scilifelab.13567355.v2.

b) If there are no restrictions, please upload the minimal anonymized data set necessary to replicate your study findings as either Supporting Information files or to a stable, public repository and provide us with the relevant URLs, DOIs, or accession numbers. For a list of acceptable repositories, please see <http://journals.plos.org/plosone/s/data-availability#loc-recommended-repositories>.

We will update your Data Availability statement on your behalf to reflect the information you provide.

The dataset is available on reasonable request at https://doi.org/10.17044/scilifelab.13567355.v2.

Reviewer #1: The authors previously published a similar study cited in reference 20 in September of 2020 showing health care workers (HCW) have a higher risk of contracting COVID-19. Here these authors essentially repeated the same serological assays and concluded quickly that this is a 4-month follow-up study to show anti-spike antibody titers were sustainable whereas anti-NP antibody titers were not.

I think the conclusion was very limited in the sense that this type of study reflected a research attitude of " salami slicing" which means that an tactic is used to provide fragmented data with a trade-off between quality and quantity of the researches. One may wonder that given the time frame now it has been more than one year since their previous report (ref 20) why these authors now report a follow-up study of only 4 months?
In addition, the conclusion brings in little new insight since nowadays more important question is to address cross-protection of antibody toward new emerging strains of virus of concern, such as delta variant and other questions alike.

We acknowledge that the results in the manuscripts are sub-analyses of a large study, and results have been published at several follow-ups (PMID: 34459525, PMID: 34391088, PMID: 33825846, PMID: 33033249). However, the data set is large and there are many aspects and findings worth reporting. Although the current manuscript was drafted several months ago, delays have prevented us from publishing until now. Nevertheless, we strongly believe the results are still very relevant considering the global spread of the virus. Published data constitute parts of the whole puzzle, and also data which corroborate findings form other studies are crucial. Furthermore, this manuscript focuses on the association between specific symptoms and disease severity, which is not within the scope of our other publications. The study period in this manuscript is also during a time with very limited spread in Sweden, reducing the effect of possible re-exposure on the association of antibody persistence and acute symptomatology. We also focus on the persistence of different specificities of anti-SARS-CoV-2 IgG in connection to symptoms and neutralization capacity, which has not been the focus at other follow-ups. We therefore believe that the data presented in this manuscript enhances and builds on data already published on this cohort, rather than corroborating already published findings.

Reviewer #2: The authors describe the serological responses to asymptomatic SARS-CoV-2 infection, indicating that, similar to symptomatic infection, antibodies against the S glycoprotein, including those with neutralising potential, remain at higher levels longer than antibodies against the nucleoprotein. Furthermore, the magnitude of antibody responses appeared to be lower in asymptomatic infection compared to symptomatic infection.

The objectives of the study are clearly described, the work is thorough, and the data are well well-described and support the conclusions drawn. Whilst these findings are not entirely novel, they do increase understanding of humoral responses in mild/asymptomatic infection. The authors do well to highlight that the asymptomatic and symptomatic cohorts differ widely, and so no direct comparisons can be made.

I like this study in its present form and have only a few minor suggestions.

1 – In the introduction, ‘Cross-sectional clinical, demographic and serological data of the HCW at study inclusion has been presented elsewhere [20]’. Would a brief description be appropriate here?

Thank you for this valuable suggestion. We have added a brief description there.

2 – in results, figure 3 – could the assay cut-offs be added to graphs to clearly depict positivity/negativity? One anti-S negative (orange) appears to have a higher anti-S IgG read out than some of the anti-S positives.

This is an important point. As described in the Methods, the cutoff is calculated as mean + 6sd of the negative controls on a per-assay-plate basis. This is done to minimize the effect of any inter-assay variations. Therefore, the cut-off will vary slightly between assay plates, and is not readily visualized as one single line. In our opinion, adding several cut-off lines in the plot would reduce rather than increase clarity.

4 – The symbols and colours use in the figures could possibly be improved to make the data more accessible.

We have improved the colors and shapes to improve readability and accessibility.
